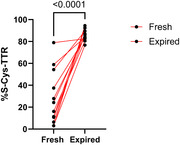# S‐Cysteinylated Transthyretin in Cerebrospinal Fluid as a Biomarker of Specimen Integrity

**DOI:** 10.1002/alz.087617

**Published:** 2025-01-09

**Authors:** Schuyler Phillip Kremer, Chad Borges

**Affiliations:** ^1^ Arizona State University, Tempe, AZ USA

## Abstract

**Background:**

Currently there is no way to determine if archived cerebrospinal fluid (CSF) specimens have been properly handled and can be considered suitable for research purposes. Transthyretin (TTR) is abundant in CSF and undergoes a redox reaction that shifts its native proteoform into an S‐cysteinylated form. This reaction proceeds spontaneously ex vivo when CSF is thawed, but ceases at storage temperatures of ‐80°C. The relative abundance of these TTR proteoforms can be measured using dilute‐and‐shoot, intact protein liquid chromatography/mass spectrometry (LC/MS). Here we report that the spontaneous ex vivo changes in TTR S‐cysteinylation that occur when CSF is in the thawed state are systematic and substantial enough to potentially qualify the relative abundance of S‐cysteinylated TTR as an endogenous marker capable of tracking specimen integrity.

**Method:**

Twelve CSF samples that were recovered postmortem were provided by Banner Sun Health Research Institute. The specimens were from patients diagnosed with Alzheimer’s or Parkinson’s disease or deemed by a pathologist as neurologically normal. A 1:2 dilution of 10 µL of CSF with 0.1% trifluoroacetic acid was directly injected onto a capillary LC system through a protein captrap (via a 10‐minute run), then into a time‐of‐flight mass spectrometer. Raw mass spectra were averaged over the TTR elution period. Following charge state deconvolution, the peak heights representing S‐cysteinylated TTR and native TTR were determined. Specimens were then incubated at 37°C for 24 hours to drive the ex vivo oxidation process to its conclusion and the LC/MS measurement process was repeated.

**Result:**

The mean relative percentage of all initially measured CSF specimens was 23.1% (± 20% SD). Following 24 hours of incubation at 37°C, the mean relative percentage of S‐cysteinylated TTR increased to 86.0% (± 5.3% SD); paired t‐test p<0.0001 and ROC c‐statistic=0.99.

**Conclusion:**

The dynamic range observed in the relative percentage of S‐cysteinylated TTR between nominally fresh and fully ex vivo‐oxidized CSF specimens was substantially greater than that observed in our previously developed ΔS‐Cys‐Albumin assay, which is used to forensically track the exposure of blood plasma and serum to thawed conditions. This suggests that a ΔS‐Cys‐TTR assay could find similar utility for archived CSF specimens.